# Nanodesigner: resolving the complex-CDR interdependency with iterative refinement

**DOI:** 10.1186/s13321-025-01069-2

**Published:** 2025-08-07

**Authors:** Melissa Maria Rios Zertuche, Şenay Kafkas, Dominik Renn, Magnus Rueping, Robert Hoehndorf

**Affiliations:** 1 Biological and Environmental Science and Engineering (BESE) Division, King Abdullah University of Science and Technology, 23955-6900 Thuwal, Saudi Arabia; 2 KAUST Beacon Development, King Abdullah University of Science and Technology, 23955-6900 Thuwal, Saudi Arabia; 3 KAUST Catalysis Center (KCC), Division of Physical Sciences and Engineering, King Abdullah University of Science and Technology, 23955-6900 Thuwal, Saudi Arabia; 4 KAUST Center of Excellence for Smart Health (KCSH), King Abdullah University of Science and Technology, 23955-6900 Thuwal, Saudi Arabia; 5 KAUST Center of Excellence for Generative AI, King Abdullah University of Science and Technology, 23955-6900 Thuwal, Saudi Arabia; 6 Institute for Experimental Molecular Imaging (ExMI), University Clinic, RWTH Aachen, Forckenbeckstraße 55, 52074 Aachen, Germany; 7 SDAIA-KAUST Center of Excellence in Data Science and Artificial Intelligence, King Abdullah University of Science and Technology, 4700 King Abdullah University of Science and Technology, Thuwal, Saudi Arabia; 8 Computer, Electrical and Mathematical Sciences and Engineering (CEMSE) Division, King Abdullah University of Science and Technology, 23955-6900 Thuwal, Saudi Arabia

**Keywords:** Nanobody design, Generative AI, Antibody design, Expectation maximization

## Abstract

Camelid heavy-chain only antibodies consist of two heavy chains and single variable domains (VHHs), which retain antigen-binding functionality even when isolated. The term “nanobody” is now more generally used for describing small, single-domain antibodies. Several antibody generative models have been developed for the sequence and structure co-design of the complementarity-determining regions (CDRs) based on the binding interface with a target antigen. However, these models are not tailored for nanobodies and are often constrained by their reliance on experimentally determined antigen–antibody structures, which are labor-intensive to obtain. Here, we introduce NanoDesigner, a tool for nanobody design and optimization based on generative AI methods. NanoDesigner integrates key stages—structure prediction, docking, CDR generation, and side-chain packing—into an iterative framework based on an expectation maximization (EM) algorithm. The algorithm effectively tackles an interdependency challenge where accurate docking presupposes *a priori* knowledge of the CDR conformation, while effective CDR generation relies on accurate docking outputs to guide its design. NanoDesigner approximately doubles the success rate of de novo nanobody designs through continuous refinement of docking and CDR generation.

## Scientific contribution

We developed a novel method for the design and optimization of nanobodies using generative AI. We use an iterative approach to address the problem that design of CDRs relies on knowledge of a complex consisting of nanobody and protein target, and accurate prediction of the complex relies on knowledge of the CDRs. We demonstrate that our method improves over the state of the art by direct comparison.

## Introduction

Antibodies are key components of the adaptive immune system, responsible for recognizing and neutralizing antigens. Structurally, they are Y-shaped proteins with two identical heavy and light chains, each containing variable and constant domains. Camelid heavy-chain only antibodies consist of two heavy chains and single variable domains (VHHs) [1], which retain antigen-binding functionality even when isolated. The term “nanobody”, trademarked by the company Ablynx (Ghent, Belgium), is now more generally used for describing small, single-domain antibodies. Due to their high specificity and low immunogenicity, these immunoglobulins are important in research and therapeutic applications [2]. Their characteristics are largely attributed to the unique structural features of the variable domains, composed of four framework regions and three hypervariable complementarity-determining region (CDR) loops. Collectively, these loops form the paratope—the site that interacts with the antigens. While most CDRs adopt constrained canonical conformations, the third CDR loop of the heavy chain (CDRH3) stands out for its high mutability. This variability significantly enhances the diversity of the antibody repertoire, enabling it to bind a wide variety of antigens [3].

Since the regulatory approval of the first monoclonal antibody (mAb) therapy in 1986, over 100 antibody therapies have been introduced globally [4]. In contrast, only four VHH-based drugs have been approved since the first one in 2019 [2]. Nanobodies may offer significant advantages, including enhanced solubility, tissue penetration, and the ability to be produced in a wider range of expression systems, all while effectively targeting similar epitopes as conventional antibodies. Their smaller paratope is compensated by greater structural diversity, especially in the CDRH3 region, which is typically 3 to 4 residues longer than in conventional antibodies [5, 6]. Additionally, an extra disulfide bond between the CDRH1 and CDRH3 regions of the VHHs enhances rigidity and stability compared to conventional antibodies [7]. These structural features enable VHHs to maintain their functionality under harsh conditions, such as high temperatures and denaturant environments, making them effective in situations where traditional antibodies might lose activity, broadening their range of potential applications [7].

Most drug candidates in clinical trials are still developed using traditional in vitro techniques, which are time-consuming and resource-intensive [8]. The increasing availability of antibody-specific data has led to the development of bioinformatics tools for rational antibody design [9]. While comprehensive computational workflows for antibody design and optimization have been reported as well, and have even been designed automatically based on interacting agents based on large language models (LLMs) [10], they often depend on computationally demanding methods such as energy minimization and molecular dynamics [11–14]. These approaches may struggle with local optima and constrained search spaces, primarily due to the extensive sampling required. The search space for a CDR of length *L* is vast, with up to $20^L$ possible sequences, making exhaustive exploration impractical. To navigate this space efficiently, generative machine learning models have been developed to learn patterns from large datasets, offering improved accuracy and efficiency in predictions while reducing computational overhead [15–18].

Generative models for antibody design initially relied on large language models trained on extensive protein sequence datasets to suggest potential mutations [19, 20]. However, these approaches are limited by their inability to incorporate essential structural information, which is crucial for accurately modeling the 3D nature of proteins. To address this limitation, methods based on Graph Neural Networks (GNNs) integrate both sequence and structural data by representing the entire protein complex (heavy, light, and antigen chains) as a graph [21], effectively capturing spatial and relational dynamics that enhance binding predictions in antibody design. Early efforts to leverage GNNs [17] began by conditioning the CDRH3 design on the antibody framework region, later expanding to include interactions with the epitope [18], albeit only considering the CDRH3 loop and ignoring the rest of the antibody structure. Subsequent advancements introduced equivariant GNNs that account for the rotational and translational symmetries inherent in protein structures, achieving state-of-the-art performance [22]. Similarly, diffusion-based models have advanced the field by enabling the co-design of CDR sequences and their corresponding structures, conditioned on the entire antibody–antigen complex, while also incorporating information about the orientation of the amino acids side chains within the binding interface [15, 23]. Reflecting the growing interest in nanobody design, concurrent work has fine-tuned RoseTTAFold Diffusion (RFDiffusion) for single-domain antibody generation [24], demonstrating the adaptability of diffusion models to this subclass of antibodies. A recently published review explores recent computational advancements in nanobody research, their impact on antigen-binding and conformational dynamics, and presents a practical example of improving a nanobody-based immunosensor, outlining future directions in healthcare and diagnostics in more detail [25].

Methods trained on antibody–antigen complex structures aim to learn the interactions within binding interfaces, theoretically enabling the de novo design of antibodies targeting unseen antigens. However, most generative methods for antibody design, including those adapted for nanobody generation, do not explicitly address the structural interdependencies between docking and CDR design. The generative CDR design process assumes prior knowledge of the antibody–antigen complex, which, when unavailable, is typically derived through computational docking. Yet, generating protein complexes via docking requires knowledge of the antibody’s structure, including the CDR. This interdependency—where CDR design relies on the complex, and complex generation depends on the CDR—remains an unresolved challenge in both antibody and nanobody design.

**Fig. 1:**
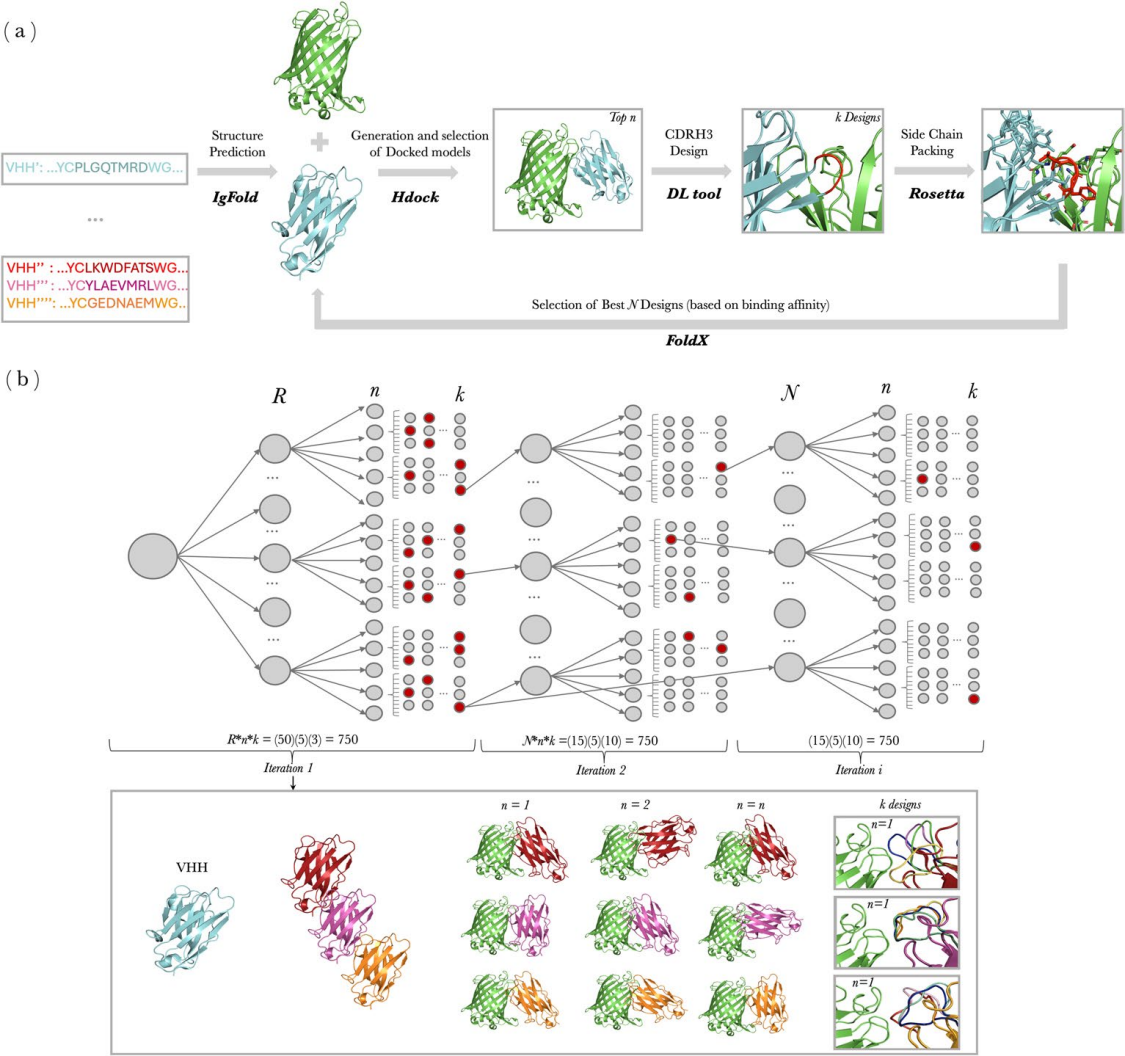
NanoDesigner workflow for the de novo design and optimization of nanobodies. **A** shows the key workflow components; additional processing steps are included between stages to ensure the quality and integrity of the intermediate inputs and final designs (see “Data quality control” section). **B** displays the variable values and total number of designs across iterations

We developed **NanoDesigner** (see Fig. 1), a novel method designed for the de novo design as well as the optimization of nanobodies. NanoDesigner integrates key stages in nanobody design, including structure prediction, docking, CDR generation, and side-chain packing. While similar workflows for antibody design are linear, we use a form of expectation maximization (EM) [26] to address the challenge that CDR design depends on accurate complexes and generating complexes requires a CDR. Our algorithm specifically improves the precision of CDRH3 loop predictions and maximizes the likelihood for identifying high-affinity, diverse nanobody candidates, over several baselines. NanoDesigner is freely available at https://github.com/bio-ontology-research-group/NanoDesigner.

## Methods

### Datasets

The data used in this study was retrieved from the Structural Antibody Database (SAbDab) in February 2024 [27]. The initial dataset included 6388 antibody–antigen complexes, and 1455 VHH antibody–antigen complexes. We filtered the entries to retain those with a resolution below 4 (Å) and targeting protein or peptide antigens. To further refine the dataset, we preserved structurally distinct binding interfaces (specifically those exhibiting CDRH3–antigen interactions) within each PDB entry, ensuring diverse interaction patterns for robust model training. “Data quality control” section describes details on how we determined the binding interface. The expanded datasets were then partitioned using two clustering strategies to address critical aspects of antibody modeling: structural diversity in CDRH3 loops [17, 28] and generalizability across antigen targets. We first clustered the sequences using MMseqs2 [29] with different sequence similarity thresholds, evaluated using the BLOSUM62 substitution matrix [29]. The clusters were then split into 10-folds. For 10-fold cross-validation, we trained on eight folds, used one fold for validation, and one for testing, iterating for 10 times and using all 10 folds for testing once; performance results are averaged across the 10 folds. For some experiments, we did not perform cross-validation due to computational limitations and instead used a single 8/1/1 split (based on the clustering results) for training, validation, and testing. Further details on the dataset analysis appear in Supplementary Materials Section S1, while clustering criteria for training and evaluation are detailed in Supplementary Materials Section S2.

### Structure prediction, docking, and CDR design

NanoDesigner follows a modular architecture comprising key components currently employed in antibody design and optimization studies [16, 30]. For structure prediction, we employed IgFold which is faster than AlphaFold and supports nanobody modeling [31]; although methods like AlphaFold 3 [32] would likely provide substantially better performance, it is not available as a stand-alone tool to be incorporated in our workflow. For docking, we chose HDOCK [33] for its efficiency in sampling thousands of models rapidly and its ability to integrate binding site information.

For CDR design, we evaluated and applied several methods. ADesigner [34] leverages graph neural networks and represents the CDR as $\mathcal {R} := \left\{ (s_i, x_{i,\omega }) \mid i = l + 1, \ldots , l + m \right\}$, where each residue is denoted by it type $s_i$, one of the 20 standard amino acids, and its backbone atom coordinates $x_i, \omega \in \ \mathbb {R}^3$ for the $\text {C}_{\alpha }$, N, C, O atoms. DiffAb [30], on the other hand, is a diffusion model that allow atomic-resolution antibody design. It achieves this by modeling each residue by its type, coordinates of the $\text {C}_{\alpha }$ atom and side-chain orientations, denoted as $s_i$, $x_i \in \mathbb {R}^3$, $O_i \in SO(3)$, respectively. The CDR is initialized arbitrarily, $\mathcal {R} := \left\{ (s_j, x_j, O_j) \mid i = l + 1, \ldots , l + m \right\}$, and the model aggregates information from the antigen and the antibody structure, then each amino acid is updated iteratively. In addition to these methods, we considered dyMEAN [16], an end-to-end deep learning tool capable of performing structure prediction, docking, and CDR generation simultaneously. It achieves full-atom design by handling residue-specific representations in a graph, where each residue is a combination of its type $s_i$ and a multi-channel 3D coordinate matrix $X_i \in \mathbb {R}^{3 \times c_i}$, where $c_i$ denotes the number of atoms. dyMEAN incorporates structural initialization and a shadow paratope to guide the docking process, aiming to improve the accuracy of epitope-binding CDR design.To modify these tools for nanobody design, we made several adjustments. The original input representation in dyMEAN and ADesigner assumed the presence of a light chain. To address this, we adjusted these methods to allow them to handle nanobody structures as well. We make all modifications available on our Github repository at https://github.com/bio-ontology-research-group/NanoDesigner.

As last step, since DiffAb and ADesigner focus only on backbone structures, we perform side-chain packing with Rosetta [35].

### Data quality control

For both input and generated data, ensuring the involvement of functional residues in binding, as well as their structural plausibility are critical for accurate antibody and nanobody design. To determine the interacting residues in nanobody– or antibody–antigen complexes, we first extracted the sequence and positional information of the CDRs using the ImMunoGeneTics (IMGT) numbering scheme [36]. This information was then used as input for differential Solvent Accessible Surface Area (dSASA) computations [37], enabling the identification of antigen residues interacting with each of the three CDRs.

To assess structural reliability of the complexes, we quantified both intra- and inter-chain steric clashes, following the approach in [38]. When clashes are detected, we performed refinement through molecular dynamics simulations and energy minimization using the AMBER99 force field within the OpenMM toolkit [39]. This iterative process reduces steric clashes and optimizes molecular geometry by adjusting atomic positions to achieve minimized potential energy.

### Evaluation scores

To quantitatively assess the structure and sequence of the generated CDR and the resulting complexes, we use amino acid recovery (AAR), which quantifies the overlap ratio between predicted and actual amino acid sequences, and root mean square deviation (RMSD), which measures structural deviation by comparing the absolute coordinates of the $\text {C}_{\alpha }$ atoms in the CDR region after Kabsch alignment [40]. Additionally, we used the TM-score [41] and local distance difference test (lDDT) [42] to evaluate global and local structural similarity, respectively, with TM-score focusing on overall topological similarity and lDDT assessing atom-level distance differences. We also use DockQ [43] to evaluate docking quality; DockQ combines scores such as interfacial contact preservation, ligand RMSD, and interface RMSD. DockQ is sensitive to the precise orientation and positioning of the antibody relative to the epitope. Additionally, we calculated differential and relative binding free energy, $\Delta G$ and $\Delta \Delta G$, using the FoldX software suite [44], to determine interaction energies and changes in binding affinity due to mutations or perturbations (in *kcal*/*mol*). Finally, we also report the success rate, defined as the percentage of designs with a negative $\Delta \Delta G$ value. This score quantifies how many designs resulted in improved binding affinity compared to a reference complex. All results are reported as the mean $\pm$ a margin of error, corresponding to half the width of the 95% confidence interval, $1.96 \times \left( \frac{\sigma }{\sqrt{n}} \right)$.

## Results and discussion

### CDR design for nanobodies

Most existing antibody CDR design tools are trained and tested on light-chain-containing antibodies or mixtures of different types of antibodies, which may limit their applicability to nanobodies. To address this limitation, we developed both a nanobody-specific dataset and an extended dataset that includes both nanobodies and conventional antibodies (see “Datasets” section). We then generated clusters for both datasets based on CDRH3 and antigen sequences at different similarity thresholds, ensuring the creation of training, validation, and test sets with no overlap between training and testing data, where each cluster contains structurally and functionally distinct molecules. We then systematically evaluated three distinct training paradigms for each clustering configuration: models trained exclusively on nanobodies, models trained on combined nanobody-antibody datasets, and models pre-trained on antibodies then fine-tuned on nanobodies. We evaluated the results based on an 8/1/1 training/validation/testing split to efficiently screen this extensive parameter space. Comprehensive results across all training configurations and threshold levels are shown in Supplementary Materials Tables S2 and 3. The results show that the best-performing training configuration combines nanobody and antibody data, clustered by antigen at 60% sequence identity, yielding the highest success rates across configurations.

For model evaluation, we performed 10-fold cross-validation (with cluster-based fold partitioning) focusing on DiffAb and ADesigner. dyMEAN demonstrated comparatively lower performance with relatively high structural clash rates, elevated binding affinity values, and high variability in energy scores, resulting in substantially lower success rates. Due to these limitations stemming from potential overfitting issues, dyMEAN was excluded from further validation. These findings align with recent work [45] which similarly identified limitations in dyMEAN’s antibody design capabilities. In addition to the optimal clustering technique and similarity threshold, we also report CDRH3 sequence recovery at 40% identity, consistent with and comparable to established benchmarks [17, 28]. Table 1 presents the results of our experiments based on the selected training and dataset configuration.

**Table 1:** Assessment of CDR antibody design tools for nanobody CDRH3 sequence and structure co-design using 10-fold cross-validation

	Tool	AAR H3 $\uparrow$	RMSD $\downarrow$	RMSD CDRH3 $\downarrow$	TMscore $\uparrow$	LDDT $\uparrow$	DockQ $\uparrow$	$\Delta G$ $\downarrow$	$\Delta \Delta G$ $\downarrow$	Clashes $\downarrow$	Success rate % $\uparrow$
Nano + antibodies											
CDRH3	ADesigner	0.385 ± 0.012	1.078 ± 0.028	2.415 ± 0.054	0.966 ± 0.002	0.831 ± 0.003	0.747 ± 0.008	8.280 ± 1.196	15.566 ± 1.228	4 ± 2	7.7 ± 1.7
	DiffAb	0.238 ± 0.007	3.664 ± 0.149	9.455 ± 0.331	0.915 ± 0.005	0.791 ± 0.006	0.278 ± 0.012	17.972 ± 3.478	25.084 ± 3.493	13 ± 4	10.8 ± 2.1
Antigen	ADesigner	0.373 ± 0.014	1.061 ± 0.027	2.418 ± 0.056	0.967 ± 0.002	0.833 ± 0.003	0.752 ± 0.009	12.141 ± 1.572	19.190 ± 1.577	6 ± 2	7.3 ± 1.6
	DiffAb	0.230 ± 0.009	3.753 ± 0.303	5.883 ± 0.602	0.912 ± 0.006	0.783 ± 0.009	0.253 ± 0.015	11.117 ± 2.769	18.534 ± 2.892	15 ± 6	15.4 ± 3.2

Performance measures are reported as mean ± margin of error (at 95% confidence), with models trained on a combined dataset of antibodies and nanobodies. Two clustering techniques were applied: clustering by CDRH3 sequence similarity, and clustering by antigen with 60% sequence similarity, based on literature and our prior findings (see Supplementary Material Section S2)

Under antigen-based clustering at 60% similarity, the two methods demonstrated distinct performance profiles, with significant statistical differences demonstrated by Mann–Whitney U tests. ADesigner maintained consistently high structural quality metrics, achieving significant advantages in AAR H3, RMSD, TMscore, LDDT, and DockQ scores, along with fewer atomic clashes (all $p < 0.001$). However, DiffAb demonstrated better design capability by generating antibodies with significantly more favorable binding energies, achieving substantially higher success rates (15.4% vs. 7.3%, $p < 0.001$), indicating its enhanced ability to produce designs with improved binding properties. This performance pattern indicates that, while ADesigner excels at structural reconstruction and maintaining geometric accuracy through its graph-based architecture, DiffAb’s diffusion-based approach generates more diverse designs that deviate further from ground truth structures but exhibit superior binding properties.

The choice of clustering strategy significantly impacts antibody design method performance, revealing distinct patterns in binding enhancement capability while maintaining consistent structural quality rankings (Table 1). Under CDRH3-based clustering (40% similarity), ADesigner achieved the best performance across nearly all metrics; it is better in all structural metrics ($p < 0.001$) and has a modest advantage in success rate for binding improvement (7.7% vs 10.8%, $p = 0.025$), but with substantial binding energy differences that lacked statistical significance ($p = 0.256$). In contrast, under antigen-based clustering (60% similarity), the performance comparison changes with DiffAb achieving significantly higher success rates (15.4% vs 7.3%, $p < 0.001$) while ADesigner retained its structural advantages. These findings indicate that antigen-based clustering creates conditions that better highlight DiffAb’s design enhancement capabilities, while CDRH3-based clustering favors ADesigner’s structural reconstruction strengths.

While our results demonstrate that CDR design methods intended for antibodies can design nanobody CDRH3 regions with a performance comparable to those reported in the antibody literature, additional challenges arise when integrating them into a complete design workflow. In particular, the input to the CDR design methods is a complex consisting of the antigen and the nanobody (or antibody). These complexes can be obtained through experimental methods or they can be generated computationally. Computational generation requires knowledge or prediction of the structure of the antigen and the nanobody (including its CDRH3 region). However, design of a CDRH3 will depend on the complex in the sense that, if the complex is different (e.g., if the nanobody binds at a different epitope), the designed CDRH3 will also be different. This leads to a mutual dependency: the design of the CDRH3 region depends on the complex, and computational generation of the complex depends on the nanobody structure (in particular its CDRH3 region at which it binds).

### NanoDesigner: end-to-end design and optimization

We developed **NanoDesigner**, an algorithm that addresses the mutual dependency between protein complex generation and CDRH3 design using Expectation-Maximization (EM) [46]. The algorithm consists of iterating multiple times between two steps: in the E-step, we generate a candidate protein complex given the current best guess of a CDRH3 region; in the M-step, we design or optimize a CDRH3 region given the generated complex. This EM process is further enhanced with: (1) beam search, which retains the top $n$ candidates at each iteration, (2) quality-controlled filtering, encompassing steric-clash resolution and binding-interface validation (“Data quality control” section), (3) and affinity-based selection, which leverages FoldX-computed binding energies to prioritize designs with superior predicted binding affinity [44]. Full algorithmic details appear in Supplementary Materials Section S3.

NanoDesigner takes as inputs the structures of a nanobody as scaffold, an antigen, and the epitope information. During the initialization step, the VHH sequence is extracted, and since the CDRH3 is unknown at this stage, we randomize it *r* times to generate *r* VHH sequences with a random CDRH3. NanoDesigner then predicts the structure of each perturbed sequence using IgFold [31].

In the E-step of the algorithm, each of the *R* predicted structures is docked with the antigen structure using HDOCK [33] to generate protein complexes. The location of the epitope and the paratope are provided as additional information to guide the docking process. For each of the randomized nanobodies, HDOCK generates *d* complexes. NanoDesigner then assesses each of these complexes for atomic clashes [38] and determines interacting residues [37].

We remove all complexes that contain clashes after refinement, and models where the CDRH3 or the epitope residues are not involved in the interactions (see “Data quality control” section). NanoDesigner then ranks all remaining complexes based on epitope recall, using the measure $\frac{|E_{\text {original}} \cap E_j|}{|E_{\text {original}}|}$ (where $E_{\text {original}}$ represents the desired epitope, i.e., the input to NanoDesigner, and $E_j$ represents the predicted epitope in the generated complex). The top $n$ docked models are then selected to move forward in the process.

In the M-step of the algorithm, a generative model (either DiffAb or ADesigner) is employed to generate $k$ designs with novel CDRH3 loops for each nanobody complex. After this step, NanoDesigner has generated a total of $r \times n \times k$ nanobodies, each in complex with an antigen, and each with a designed and optimized CDRH3. These mutants then undergo side-chain packing using the Rosetta software [35]. The designed nanobodies are then ranked based on the predicted binding affinity energy $\delta$ computed with FoldX [44], with stronger binding affinities receiving higher rankings. We retain the top-*n* ranking nanobodies and use them as input to the next E-step. Only in the first iteration, we select the top-*n* from all *r* lineages, i.e., we keep the top-ranking nanobody from each of the original *r* (randomized) lineages; in subsequent iterations, we rank all designed nanobodies by binding affinity and keep the top-*n* across all. The reason for keeping the best performing nanobody from each lineage in the first iteration is that we found that binding affinities generally improve more in the second iteration, and keeping each lineage allows us to maintain higher diversity. Figure 1 gives an overview of NanoDesigner.

There are two applications for nanobody CDRH3 design: optimizing the CDRH3 of an existing nanobody with known binding to an antigen (“nanobody optimization”) and designing a CDRH3 de novo without any prior information about a complex. NanoDesigner can be used both for nanobody optimization and de novo design, by modifying only the final ranking based on the measure $\delta$. In nanobody optimization, where a complex is already known, the algorithm optimizes the predicted relative binding free energy ($\Delta \Delta G$), with the objective to reduce this energy when compared with the reference complex. This approach is useful for enhancing the binding affinity of an existing nanobody, such as one targeting a mutating antigen. In de novo design, where no complex is known, the focus is on optimizing the predicted absolute binding free energy ($\Delta G$) which is valuable for targeting novel antigens. We compared NanoDesigner with a linear design workflow without the iterative EM loop as a baseline; results are shown in Table 2.

**Table 2:** NanoDesigner in the nanobody optimization and de novo scenarios

Score	DiffAb				ADesigner			
	Optimization		De novo		Optimization		De novo	
	Baseline	Ours	Baseline	Ours	Baseline	Ours	Baseline	Ours
AAR H3 $\uparrow$	0.069 ± 0.009	0.066 ± 0.011	0.076 ± 0.007	0.074 ± 0.008	0.327 ± 0.030	0.534 ± 0.091	0.248 ± 0.031	0.323 ± 0.041
RMSD $\downarrow$	3.76 ± 0.30	3.29 ± 0.38	3.93 ± 0.19	4.37 ± 0.21	0.93 ± 0.05	0.88 ± 0.12	1.32 ± 0.20	0.92 ± 0.06
RMSD CDRH3 $\downarrow$	4.21 ± 0.17	4.48 ± 0.34	4.24 ± 0.16	4.62 ± 0.21	2.14 ± 0.12	2.03 ± 0.34	2.71 ± 0.20	2.06 ± 0.13
TMscore $\uparrow$	0.860 ± 0.005	0.816 ± 0.016	0.835 ± 0.011	0.820 ± 0.012	0.951 ± 0.008	0.971 ± 0.004	0.887 ± 0.028	0.969 ± 0.002
LDDT $\uparrow$	0.658 ± 0.010	0.611 ± 0.023	0.644 ± 0.011	0.615 ± 0.015	0.822 ± 0.009	0.833 ± 0.005	0.747 ± 0.026	0.833 ± 0.006
DockQ $\uparrow$	0.097 ± 0.009	0.088 ± 0.011	0.126 ± 0.012	0.113 ± 0.012	0.730 ± 0.026	0.720 ± 0.024	0.566 ± 0.043	0.717 ± 0.022
$\Delta G$ $\downarrow$	0.184 ± 0.218	− 5.963 ± 0.271	− 0.685 ± 0.191	− 7.592 ± 0.208	2.587 ± 0.213	− 7.341 ± 0.275	3.127 ± 0.287	− 2.124 ± 0.179
$\Delta \Delta G$ $\downarrow$	6.3 ± 0.435	0.807 ± 0.461	5.95 ± 0.424	− 1.036 ± 0.384	8.6 ± 0.457	− 1.291 ± 0.518	9.65 ± 0.478	4.546 ± 0.403
Clashes $\downarrow$	0	0	0	0	0	0	0	0
Success Rate % $\uparrow$	19.692	47.592	18.692	57.287	11.019	45.135	10.877	39.074

Each pair of columns compares the average $\pm$ margin of error (at 95% confidence) for measures obtained on a test set. We compare a linear workflow of NanoDesigner without the EM loop (baseline) with the results of NanoDesigner with the EM loop for 10 iterations. The test set ($n \approx 100$ nb) is a single fold from a dataset combining antibodies and nanobodies), clustered by antigen sequence similarity at 60% (see “Datasets” section)

Our results demonstrate that NanoDesigner consistently and substantially improves over the linear design workflow, demonstrating that our iterative refinement approach is successful: NanoDesigner achieves a significantly higher success rate, more than twice compared to the baseline linear workflow, and achieves, for example, 57% success rate using DiffAb and 39% using ADesigner for CDRH3 design. This improvement is driven by iterative optimization, which refines nanobody designs and enhances binding affinities, as shown in Supplementary Material Figure S5, where $\Delta \Delta G$ values steadily improve across iterations. Although NanoDesigner shows slightly lower performance on CDRH3 design scores such as AAR, TMScore, LDDT, and DockQ (Table 1), it substantially improves binding energy. The improved $\Delta G$ and $\Delta \Delta G$ values suggest that greater diversity in the CDRH3 region drives stronger affinities. By exploring a wider range of conformations, NanoDesigner enhances binding and reduces the likelihood of clashes, leading to more effective interactions with the target antigen. Similar trends were observed in the de novo design scenario, with NanoDesigner showing consistent improvements in binding affinity compared to the baseline. As shown in Table 2, our method enhances success rates and binding energetics, demonstrating its robustness across both optimization and de novo design tasks.

Analysis on the sequence diversity of the generated designs for each CDR design methods revealed that ADesigner generated sequences with tendency to include repetitive amino acids, while DiffAb exhibited the highest sequence variability, suggesting broader exploration of the design space (Supplementary Material Figure S6). This higher sequence diversity likely contributes to NanoDesigner’s improved performance, as it enables more effective sampling of high-affinity binders.

### NanoDesigner: test cases

Based on our findings, we employed NanoDesigner, with DiffAb for the CDRH3 generation stage, on three distinct antigens: mNeonGreen, KRAS, and HER2, selected for their significance in research and therapeutic applications. For mNeonGreen (PDB:5LRT) and KRAS (PDB:4OBE), epitope sequences were provided by domain experts. For HER2, we identified epitope sequences via dSASA analysis using HER2’s antibody-bound structure (PDB:8PWH); in this structure, trastuzumab and pertuzumab define two distinct epitopes. The HER2 antigen chain was then isolated for use in the NanoDesigner process (see “Data quality control” section).

**Fig. 2:**
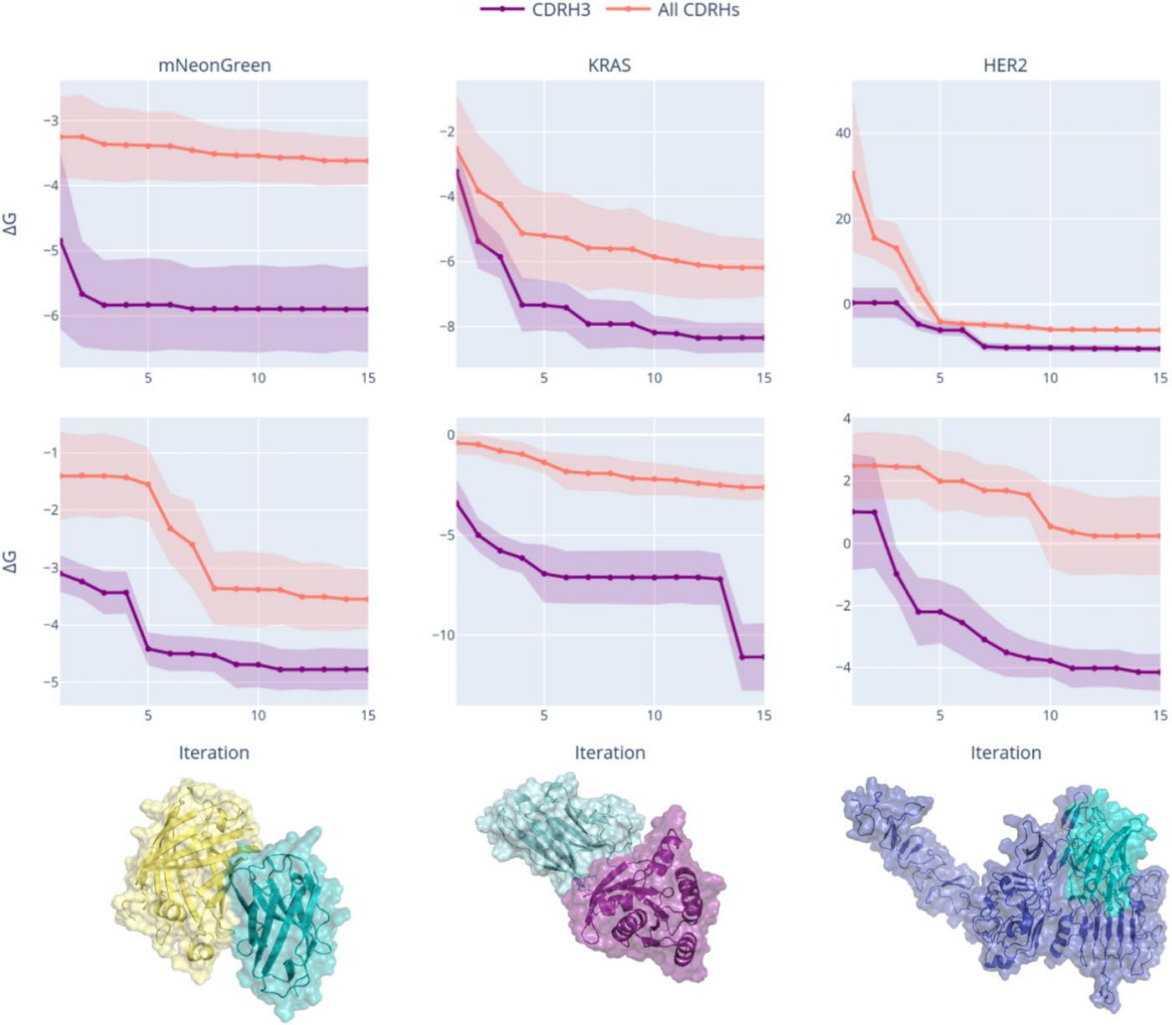
NanoDesigner for de novo design—test cases. The $\Delta G$ values for the design of CDRH3 (purple) or all CDRs (orange) are tracked across iterations for three distinct antigens: mNeonGreen (PDB:5LRT), KRAS (PDB:4OBE), and HER2 (PDB:8PWH), represented from left to right in the panels. A camelid nanobody scaffold (PDB:6LR7) was utilized in the first two panels, while a humanized nanobody scaffold (PDB:7EOW) was employed in the third panel. Within each antigen panel, two distinct epitope sites were analyzed. The first epitope is displayed at the top, while the second is shown in the bottom. At the bottom of each panel, a representative design per nanobody–antigen complex is illustrated for the first epitope. The plotted lines represent average $\Delta G$ values across epitopes, with shaded regions indicating variability. Epitope details and VHH scaffold data are available in Supplementary Table S5

Our dataset analysis revealed that all three CDRs are highly involved in binding (see Supplementary Material Figure S1, panel (b)). Therefore, to assess whether optimizing all CDRs simultaneously improves design outcomes, we trained DiffAb following the optimal training configuration for multiple CDR design (Supplementary Material Table S6). We then used NanoDesigner to design either one (CDRH3) or all three CDRs for each of the three targets. Figure 2 indicates that, when focusing only on CDRH3, the algorithm converges more quickly and with better $\Delta G$ values, and with a steeper decline in energy during early iterations compared to models that optimize all three CDRs. This observation aligns with our diversity analysis (Supplementary Material Figure S2), which revealed that CDRH3 exhibits exceptional variability in both length and sequence composition compared to CDRH1 and CDRH2. By focusing solely on CDRH3, the algorithm can efficiently explore the most diverse and functionally critical region of the paratope, whereas adding the less diverse CDRH1 and CDRH2 to the optimization significantly increases the search space without proportionally enhancing binding potential. This suggests that the added complexity of simultaneously optimizing all three CDRs may actually impede convergence rather than improve final designs. Additionally, the variability of $\Delta G$ values across our test cases suggests that some epitopes, such as those we selected in KRAS and HER2, present greater difficulty in design, likely due to more complex or variable binding interfaces. However, further investigation of epitope-specific optimization strategies would be valuable and will remain a focus of future research efforts.

## Conclusion

We developed NanoDesigner, an algorithm that improves the de novo nanobody design as well as nanobody optimization. NanoDesigner overcomes a limitation in traditional antibody design methods where generation of CDR loops using generative models is dependent on knowledge of a complex, and predicting the complex structure depends on knowing the CDR sequence. NanoDesigner also provides specific optimizations and evaluation results for nanobodies, in contrast to other methods focusing on general antibodies. NanoDesigner is suited for designing nanobodies for previously uncharacterized antigens and antigens for which neither a nanobody nor antibody is available.

As a proof of concept, our current implementation uses established tools to ensure reproducibility and comparability with state-of-the-art methods. The framework’s modular design facilitates seamless incorporation of emerging techniques (structure prediction, docking, or CDR generation) allowing newer, higher-performance tools to be integrated as they become available. This flexibility ensures continuous improvement while preserving the core methodology and maintaining reproducibility, key requirements for tool development.

## Supplementary Information

Supplementary Material 1.

## Data Availability

No datasets were generated or analysed during the current study.

## Code availability

The code supporting the findings of this study is freely available on https://github.com/bio-ontology-research-group/NanoDesigner. All data generated or analyzed in this study can be reproduced by following the procedures detailed in the link. Users can access the code and replicate the steps provided to produce the data associated with this study.
