# Supplementary material for: Nanodesigner: resolving the complex-CDR interdependency with iterative refinement
Source: J Cheminform. 2025 Aug 7;17:120. doi: 10.1186/s13321-025-01069-2 (PMC12333243; doi:10.1186/s13321-025-01069-2)
Supplement: Supplementary file 1 — Supplementary Material 1. [file 13321_2025_1069_MOESM1_ESM.pdf]

NanoDesigner: Resolving the complex–CDR interdependency with iterative refinement

Supplementary Material

Melissa Maria Rios Zertuche[1], Şenay Kafkas[2], Dominik Renn[3], Magnus Rueping[3,4,5,6], Robert Hoehndorf[1,4,5,7,8,*]

[1]Biological and Environmental Science and Engineering (BESE) Division, King Abdullah University of Science and Technology, 23955-6900, Thuwal, Saudi Arabia.
[2]KAUST Beacon Development, King Abdullah University of Science and Technology, 23955-6900, Thuwal, Saudi Arabia.
[3]KAUST Catalysis Center (KCC), Division of Physical Sciences and Engineering, King Abdullah University of Science and Technology, 23955-6900, Thuwal, Saudi Arabia.
[4]KAUST Center of Excellence for Smart Health (KCSH), King Abdullah University of Science and Technology, 23955-6900, Thuwal, Saudi Arabia.
[5]KAUST Center of Excellence for Generative AI, King Abdullah University of Science and Technology, 23955-6900, Thuwal, Saudi Arabia.
[6]Institute for Experimental Molecular Imaging (ExMI), University Clinic, RWTH Aachen, Forckenbeckstraße 55, D-52074, Aachen, Germany.
[7]SDAIA–KAUST Center of Excellence in Data Science and Artificial Intelligence, King Abdullah University of Science and Technology, 4700 King Abdullah University of Science and Technology, Thuwal, Saudi Arabia.
[8]Computer, Electrical and Mathematical Sciences and Engineering Division, King Abdullah University of Science and Technology, 23955-6900, Thuwal, Saudi Arabia.

Corresponding author email: robert.hoehndorf@kaust.edu.sa

S1 Dataset statistics and analysis

The data used in this study was retrieved from the Structural Antibody Database (SAbDab) in February 2024 [1]. The initial dataset included 6,388 antibody–antigen complexes, and 1,455 VHH antibody–antigen complexes. Instances in the dataset correspond to entries in the Protein Data Bank (PDB) [2]. The PDB entries contain structural information for all chains present, including the identifiers for heavy, light, and antigen chains, and relevant metadata such as the resolution of the structure.

Instances were filtered to retain only those with a resolution quality below 4 Å, retain nanobodies that target protein or peptide type antigens, and ensure that the heavy, light, and antigen chain identifiers were completely identified. Given that the generative models used in our work are designed to learn from interactions involving the complementarity-determining region (CDR) loops of the heavy chain, we removed all complexes that did not have interactions with the CDR loops; we calculated the delta solvent accessible surface area (dSASA) to determine the binding interface [3]. All entries were renumbered according to the IMGT scheme, which assigns specific positions based on the conserved residues in the antibody framework. This provided a standardized way to locate the CDRs, with the paratope defined as the residues comprising the CDRH1, CDRH2, and CDRH3 loops. We used dSASA calculation to generate an interaction matrix between two biomolecules and identify specific binding pairs, and we used this to compute the percentage involvement of each CDR. All filtered entries showing interaction were renumbered back to their appropriate numbering schemes to ensure compatibility with the different generative tools used in subsequent analyses. For instance, Chothia numbering [4] was applied for DiffAb.

S1.1 Antibody-Antigen complexes and Nanobody-Antigen Complexes Binding Analysis

As shown in Table S1, although nanobody-containing entries account for 1,455 unique PDBs, we observe 2,878 total nanobody–antigen binding instances, with 1,730 (approximately 60%) showing interactions involving CDRH3. In contrast, antibodies span a much larger set of 6,388 unique PDBs and 12,207 binding instances, with 6,446 (around 53%) of these involving CDRH3 interactions. This higher proportion of CDRH3 involvement in nanobodies compared to conventional antibodies reflects the structural differences between these two systems: nanobodies lack light chains and therefore rely more heavily on their heavy-chain CDRs, particularly CDRH3, for antigen recognition. Conventional antibodies, in contrast, can utilize both heavy and light chain interactions, distributing binding contributions across a broader set of complementarity-determining regions. These differences highlight distinct structural and functional constraints in antigen recognition between nanobodies and conventional antibodies—factors that are crucial to consider when modeling or predicting binding interfaces in our study.

Table S1: Dataset statistics. The number of unique PDB complexes is shown in parenthesis; total number of instances refers to different nanobody– or antibody–antigen pairs.

	**Antibodies**	**Nanobodies**
**Total PDB entries**	6388 (6388)	1455 (1455)
**Total Instances**	12207 (4296)	2878 (1145)
**Filtered instances**	8027 (4296)	2094 (1145)
**Instances interacting from the paratope**	6545 (3463)	1749 (1013)
**Instances interacting from CDRH3**	6446 (3413)	1730 (1007)

All nanobody instances retained after the data pre-processing step were analyzed to determine the lengths of CDRH1, CDRH2, and CDRH3, as well as their average involvement in interactions with corresponding antigen proteins or peptides.

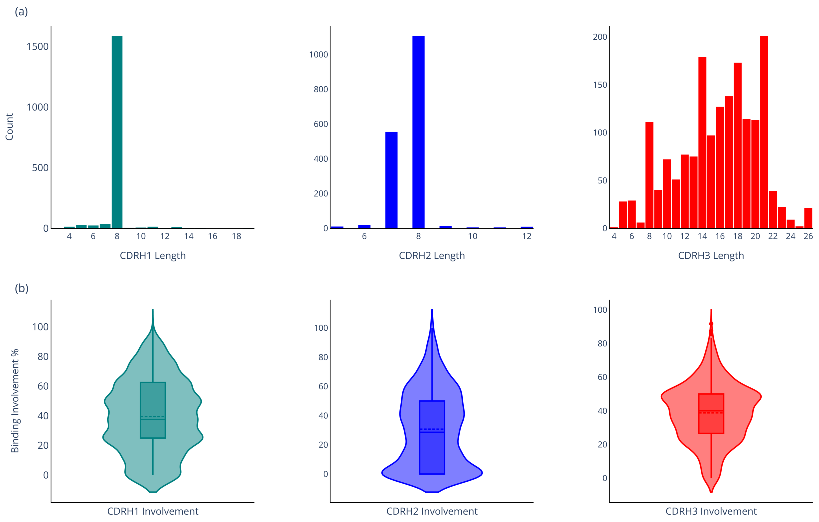

Fig. S1: Length and binding involvement of nanobody CDRHs.

Panel (a) from Figure S1 displays the length distributions of CDRH1, CDRH2, and CDRH3. The lengths of CDRH1 and CDRH2 show a clear clustering around 8 amino acids, which may be associated with their tendency to adopt canonical structures commonly observed in nanobodies. In contrast, CDRH3 exhibits a much wider range of lengths, from 4 to over 26 amino acids, indicating substantial variability. This diversity is expected, as CDRH3 plays a critical role in antigen specificity and frequently deviates from canonical shapes, allowing for greater flexibility and adaptability in binding interactions. Panel (b) in Figure S1 shows the binding involvement percentages of CDRH1, CDRH2, and CDRH3 in nanobody–antigen complexes. The violin plots highlight the variability in contribution of each CDR loop to antigen binding. CDRH1 and CDRH2 exhibit moderate involvement, with median values around 40-50%, indicating their consistent, but less dominant, roles in binding interactions. In contrast, CDRH3 shows a broader range of involvement, with a high frequency of instances near the upper bound of participation, supporting its role as the primary driver of antigen specificity. The variability in CDRH3 binding involvement aligns with its structural flexibility and longer length, emphasizing its adaptability and key function in facilitating diverse immune responses.

S1.2 Diversity Analysis of Complementarity-Determining Regions (CDRs)

The statistical comparison of CDR diversity reveals notable differences across regions. CDRH3 displays greater variability in both length ($\sigma = 4.57$) and sequence composition (mean positional entropy = 2.82) compared to the more constrained diversity observed in CDRH1 and CDRH2 ($\sigma \leq 0.86$, entropy ≤ 2.27). To assess the relative contributions of sequence versus length diversity, we examined CDRs at their modal lengths: where CDRH1 and CDRH2 typically cluster around 8 residues, CDRH3 shows a modal length of 21 residues. Even when comparing CDRH3 artificially constrained to 8 residues, it exhibits higher sequence diversity than CDRH1 ($t = -60.74$, Cohen's $d = -2.56$) and CDRH2 ($t = -62.93$, Cohen's $d = -2.65$), suggesting its variability arises from both length and sequence composition.

These observations may help explain CDRH3's prominent role in antigen binding and could inform its prioritization in optimization algorithms. The tendency of energy-minimizing designs to focus on CDRH3 (Figure S2) is consistent with its capacity to access more diverse conformations, potentially contributing to its functional importance in antibody-antigen interactions.

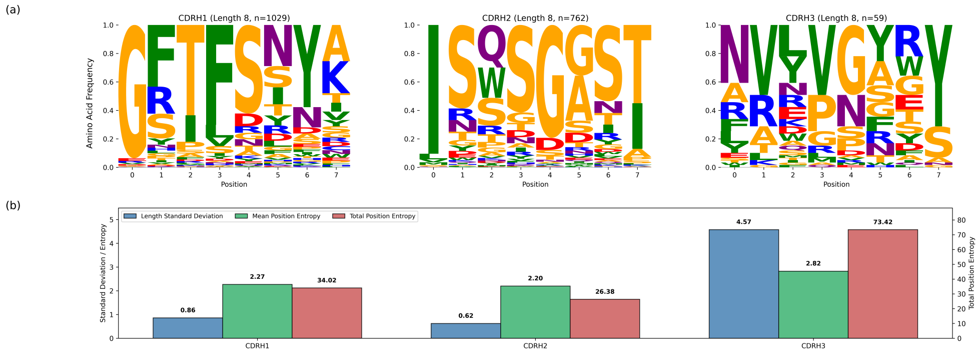

Fig. S2: Comparative diversity analysis of nanobody CDRH regions. (a) Sequence logos of CDRHs with lengths normalized to their respective modes (8 for CDRH1/2, fixed to 8 for CDRH3 for comparison). (b) Diversity metrics comparing length variation and positional entropy.

S2 Assessment of generative methods for nanobody CDRH3 design

S2.1 Selection of Dataset Clustering and Training Configuration

The partitioning strategy for our datasets was designed to address two critical aspects of antibody modeling: structural diversity in CDRH3 loops and generalizability across

antigen targets. We systematically evaluated multiple sequence identity thresholds for both clustering dimensions using MMseqs2 [5] with the BLOSUM62 substitution matrix[5], ensuring consistent comparison across all analyses.

For CDRH3 clustering, we examined thresholds spanning 20% to 40% sequence identity. The 40% threshold aligns with standard practices in computational antibody design [6, 7], maintaining sufficient structural similarity within clusters while preserving meaningful diversity between them. The inclusion of 30% and 20% thresholds allows us to examine how models perform when trained on progressively more diverse structural clusters, where sequence similarity becomes a less reliable predictor of functional similarity. This range captures the natural variation observed in CDRH3 loops while avoiding the artificial homogenization that would occur at higher identity thresholds.

For antigen sequence clustering, we employed thresholds of 60%, 80%, and 95% identity to systematically evaluate model performance across different levels of antigenic similarity. The 95% threshold creates clusters of highly similar antigens, providing a stringent test for generalization to novel targets that share minimal sequence similarity with training antigens. The 80% and 60% thresholds create progressively more diverse antigen clusters, allowing us to assess model performance when training and test antigens exhibit moderate to substantial sequence divergence. This graded approach enables us to evaluate whether models can generalize binding predictions across antigens of varying similarity levels, rather than relying on memorization of specific antigenic sequence patterns.

The clusters were then split into 10 folds. For 10-fold cross-validation, we trained on eight folds, used one fold for validation, and one for testing, iterating for 10 times and using all 10 folds for testing once; performance results are averaged across the 10 folds. For the current analysis, we did not perform cross-validation due to computational limitations and instead used a single 8/1/1 split (based on the clustering results) for training, validation, and testing.

All threshold combinations were evaluated across three training configurations using: (1) nanobody-only datasets, (2) combined nanobody-antibody datasets, and (3) antibody-trained models fine-tuned on nanobody data. This systematic comparison allows us to examine how different data compositions affect nanobody design performance across clustering thresholds. Performance was benchmarked against three leading CDR design methods: ADesigner (structure-based), DiffAb (diffusion-based), and dyMEAN (energy-aware), covering the major computational approaches to antibody design. Results are summarized in Tables S3 and S2, which provides various measures widely employed in antibody design.

Table S2: Performance Measures of Antibody Design Tools with CDRH3 Loop Clustering at Different Sequence Identity Thresholds for Nanobody Design

		Tool	AAR H3 ↑	RMSD ↓	RMSD CDRH3 ↓	TM-score ↑	LDDT ↑	ΔG ↓	ΔΔG ↓	Clashes ↓	DockQ ↑	Success Rate (%) ↑
Nb+Ab	20%	ADesigner	0.362	1.000	2.282	0.969	0.837	4.714	12.791	3	0.736	5.0
		DiffAb	0.251	3.116	8.514	0.920	0.792	15.409	24.142	26	0.284	9.0
		dyMEAN	0.437	12.545	4.523	0.910	0.772	278.786	287.520	222	0.604	0.0
	30%	ADesigner	0.387	0.932	2.229	0.973	0.845	4.598	12.672	2	0.744	8.0
		DiffAb	0.222	3.998	11.541	0.902	0.765	6.185	13.389	27	0.244	13.3
		dyMEAN	0.414	12.007	4.809	0.929	0.799	254.605	262.157	198	0.619	0.0
	40%	ADesigner	0.487	0.978	2.191	0.966	0.839	4.237	11.651	2	0.752	13.0
		DiffAb	0.274	3.266	9.272	0.920	0.811	6.447	14.496	4	0.327	12.0
		dyMEAN	0.537	9.574	3.872	0.940	0.820	191.088	199.138	141	0.661	0.0
Nanobodies	20%	ADesigner	0.231	1.331	3.056	0.951	0.804	8.757	17.492	8	0.650	6.0
		DiffAb	0.210	5.161	13.863	0.896	0.754	13.938	22.391	47	0.170	15.0
		dyMEAN	0.205	24.106	10.051	0.902	0.752	203.632	212.085	33	0.319	0.0
	30%	ADesigner	0.262	1.267	2.850	0.960	0.818	10.139	18.064	20	0.646	6.0
		DiffAb	0.236	5.158	15.487	0.804	0.645	19.330	27.032	92	0.069	12.1
		dyMEAN	0.239	24.997	11.379	0.907	0.772	227.452	235.348	25	0.303	0.0
	40%	ADesigner	0.252	1.365	3.077	0.958	0.822	5.603	16.418	2	0.607	3.0
		DiffAb	0.232	3.538	9.968	0.909	0.778	16.550	26.950	37	0.279	4.0
		dyMEAN	0.219	21.530	11.182	0.908	0.781	740.923	751.394	307	0.324	0.0
Fine-tuned	20%	ADesigner	0.219	1.163	2.452	0.952	0.808	10.057	18.793	5	0.669	5.0
		DiffAb	0.206	3.970	4.136	0.902	0.767	10.755	19.208	21	0.214	12.0
		dyMEAN	0.206	8.769	5.878	0.405	0.273	3068.266	3076.718	8437	0.294	0.0
	30%	ADesigner	0.250	1.183	2.606	0.965	0.829	11.398	19.095	12	0.688	8.0
		DiffAb	0.203	3.706	10.224	0.913	0.789	15.699	23.462	24	0.260	15.0
		dyMEAN	0.275	14.279	7.997	0.396	0.273	2470.146	2477.989	8982	0.272	0.0
	40%	ADesigner	0.227	1.301	2.883	0.962	0.827	9.829	20.699	0	0.694	3.0
		DiffAb	0.206	4.720	13.220	0.907	0.783	8.630	19.029	10	0.213	9.0
		dyMEAN	0.238	20.970	11.214	0.398	0.274	2137.379	2147.405	7026	0.254	0.0

Table S3: Performance Measures of Antibody Design Tools with Antigen Sequence Clustering at Varying Similarity Levels for Nanobody Design

		Tool	AAR H3 ↑	RMSD ↓	RMSD CDRH3 ↓	TM-score ↑	LDDT ↑	ΔG ↓	ΔΔG ↓	Clashes ↓	DockQ ↑	Success Rate (%) ↑
Nb+Ab	60%	ADesigner	0.361	1.017	2.323	0.966	0.836	7.736	13.752	1	0.745	16.0
		DiffAb	0.229	4.711	12.942	0.897	0.745	1.583	7.867	21	0.209	**21.0**
		dyMEAN	0.423	14.182	7.445	0.924	0.792	114.465	119.604	8	0.537	5.0
	80%	ADesigner	0.399	0.841	1.959	0.977	0.851	2.990	10.835	3	0.765	9.0
		DiffAb	0.234	4.843	12.812	0.883	0.722	1.916	8.423	36	0.211	15.0
		dyMEAN	0.548	10.071	3.867	0.938	0.808	142.771	149.278	23	0.685	1.0
	95%	ADesigner	0.479	1.002	2.251	0.972	0.839	10.498	16.516	5	0.767	9.0
		DiffAb	0.219	3.001	8.003	0.929	0.799	8.432	14.146	13	0.332	17.0
		dyMEAN	0.495	10.443	4.018	0.887	0.759	52.526	57.715	7	0.655	2.0
Nanobodies	60%	ADesigner	0.259	1.281	2.926	0.961	0.818	13.237	20.660	10	0.702	4.0
		DiffAb	0.220	3.680	9.831	0.909	0.785	16.180	25.181	16	0.276	4.0
		dyMEAN	0.253	25.016	10.194	0.878	0.703	544.417	553.417	179	0.349	0.0
	80%	ADesigner	0.294	1.079	2.618	0.967	0.827	7.027	13.000	6	0.658	3.0
		DiffAb	0.234	4.024	11.150	0.910	0.774	20.403	26.528	25	0.219	5.0
		dyMEAN	0.213	23.622	10.878	0.894	0.755	266.430	272.555	42	0.315	0.0
	95%	ADesigner	0.355	1.076	2.471	0.967	0.830	4.859	13.307	2	0.750	4.0
		DiffAb	0.222	36.152	73.593	0.869	0.716	13.117	21.293	37	0.176	10.0
		dyMEAN	0.279	20.725	9.113	0.861	0.721	335.721	327.111	60	0.372	0.0
Fine-tuned	60%	ADesigner	0.228	0.981	2.134	0.965	0.826	14.583	21.860	12	0.729	4.0
		DiffAb	0.259	3.043	3.872	0.916	0.798	13.232	22.233	8	0.319	7.0
		dyMEAN	0.235	9.143	6.203	0.384	0.278	3411.579	3420.579	10355	0.334	0.0
	80%	ADesigner	0.264	1.052	2.495	0.968	0.827	11.565	17.537	5	0.704	5.0
		DiffAb	0.249	7.031	13.729	0.920	0.789	13.979	20.104	21	0.251	10.0
		dyMEAN	0.301	11.710	6.687	0.400	0.276	2442.629	2449.048	7328	0.258	0.0
	95%	ADesigner	0.272	1.093	2.479	0.966	0.828	9.029	17.477	5	0.748	3.0
		DiffAb	0.219	3.605	4.315	0.900	0.769	3.333	11.510	53	0.203	12.0
		dyMEAN	0.291	19.965	9.886	0.384	0.269	3066.565	3074.741	9159	0.316	0.0

S2.2 Performance as a Function of CDRH3 Length and Analysis of Affinity Distribution

CDRH3 length-performance relationships (Figure S3) were assessed using pooled design results from Section S2.1 and Pearson correlation analysis across six metrics, with success rate defined as the proportion of designs exhibiting improved binding affinity compared to a reference complex ($\Delta\Delta G < 0$). DiffAb exhibited strong length dependence with near-perfect negative correlations for structural metrics (TMscore: $r = -0.994$, LDDT: $r = -0.980$, DockQ: $r = -0.915$; all $p < 0.001$) and moderate negative correlations for design success ($r = -0.674$, $p = 0.001$). ADesigner showed similar but weaker trends (TMscore: $r = -0.926$, LDDT: $r = -0.939$, success rate: $r = -0.567$; all $p \leq 0.007$) but no DockQ correlation ($r = 0.000$). In contrast, dyMEAN demonstrated complete length independence with no significant correlations (all $p > 0.05$). While DiffAb and ADesigner show systematic performance deterioration with longer CDRH3 loops, dyMEAN's length-agnostic behavior reflects its fundamentally different approach to sequence generation, as evidenced by its broader and more variable ΔG distribution compared to the natural dataset (Figure S4).

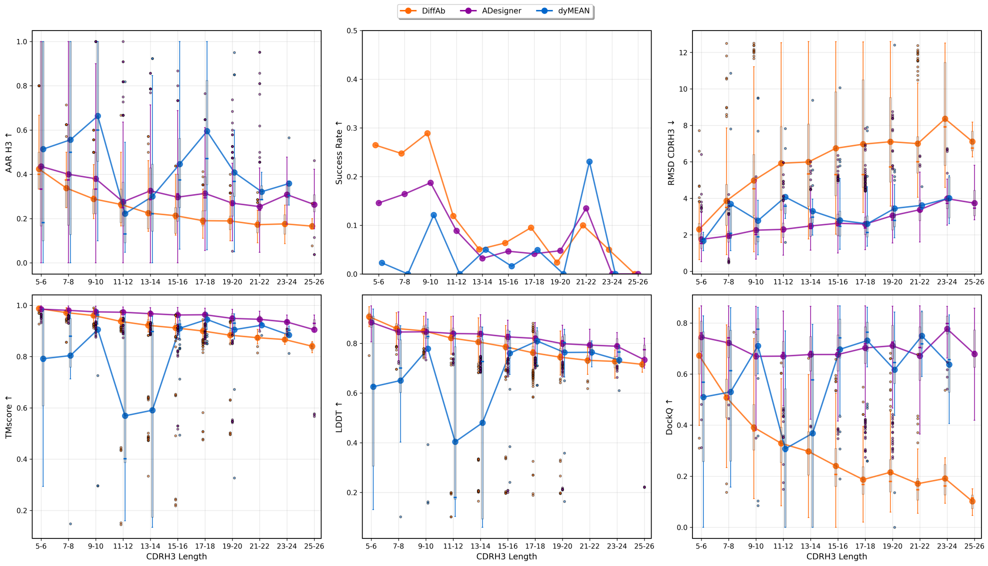

Fig. S3: Impact of CDRH3 Loop Length on Computational Nanobody Design Performance.

Figure S4 shows a comparison of the differential binding free energy (ΔG, in $kcal/mol$) distributions between the original nanobody dataset and the pooled designs generated by DiffAb, ADesigner, and dyMEAN, all evaluated using the FoldX software suite [8]. The density plots show that DiffAb and ADesigner produce designs that closely match the natural distribution of affinity energies in the dataset, whereas

dyMEAN displays a more variable distribution that does not align well with the affinity profiles of the reference dataset.

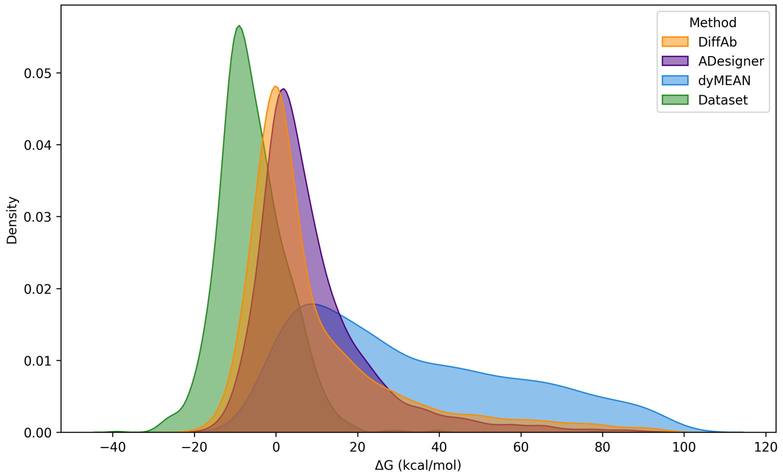

Fig. S4: Affinity distribution of natural and predicted nanobody-antigen complexes across CDR design tools.

S3 NanoDesigner algorithm description

The NanoDesigner algorithm is divided into key phases analogous to the steps in the expectation–maximization (EM) algorithm: initialization, expectation, maximization, and convergence test. In the initialization phase, the nanobody scaffold undergoes sequence randomization, with $r = 50$ sequences being generated in our experiments. This randomization focuses on either the CDRH3 or all CDRs simultaneously, serving as an initial exploration of the sequence space to generate candidates for subsequent design and optimization. For structure prediction, IgFold is used to predict 3D structures for the r sequences. Throughout the workflow, quality control checks, such as structural validation and refinement, are integrated at every stage to ensure only reliable intermediate structures proceed to subsequent steps.

During the expectation step, a set of nanobody–antigen complexes is obtained through docking simulations performed by HDOCK for each randomized nanobody sequence. The inputs include the structure of the randomized nanobody, the target antigen structure, and their respective complementary binding site information: the paratope and the epitope. The paratope is defined as the collection of the three CDRs. If a nanobody–antigen complex is provided, the epitope is identified through dSASA analysis during a preprocessing step. Otherwise, the epitope must be provided as an amino acid sequence and mapped onto the antigen structure. HDOCK with default parameters generates 100 candidate models per simulation; this number can be adjusted according to experimental requirements. The complex selection

process determines which docked models proceed to the next stage as inputs. Priority is given to complexes that exhibit high epitope recall. To maintain a balance between exploration and optimization, the algorithm employs a beam search strategy, retaining only the top n models sorted by epitope recall. This allows the algorithm to explore a diverse range of solutions while focusing on promising candidates for further refinement and optimization.

In the maximization step, for each docked model, a set of k CDR designs are generated using either the DiffAb or ADesigner methods. These designs undergo a complex selection process to filter out models based on structural quality (presence of "clashes" where atoms would overlap in space) and the involvement of the CDR loops in interactions (i.e., we remove all CDR designs where the generated CDR loop is not involved in an interaction with the antigen epitope). Only the designs that pass these quality filters proceed to the evaluation and ranking phase. The ranking is based on an objective function δ that measures binding efficiency. The function δ is either the binding free energy (ΔG) or the relative binding free energy ($\Delta\Delta G$), depending on whether the workflow focuses on *de novo* design or optimization scenarios. At the end of this step, the top n designs with the best ranking scores (based on δ are selected to proceed to the next iteration.

In all our experiments, convergence is determined by a predefined number of total iterations. This fixed iteration limit ensures that the design process terminates consistently across runs. However, alternative convergence criteria could be applied to halt the iterative process based on change in the overall evaluation metrics. The computational resources required by NanoDesigner for each stage—including runtime, memory usage, and GPU/CPU demands—are summarized in Table S4. The protocol's most resource-intensive steps are docking simulation and CDRH3 design.

Table S4: Computational resource requirements for NanoDesigner protocol. Resource usage estimates were measured on NVIDIA V100 GPU with 128 CPU cores.

**Stage**	**Time (s)**		**Entries**		**RAM (GB)**		**GPU (GB)**	
	**Iter 1**	**Iter X**	**Iter 1**	**Iter X**	**Avg.**	**Peak**	**Avg.**	**Peak**
Structure Prediction	278.6	81.8	50	15	52.5	54.5	27.4	27.4
Docking Simulation	4944	1483	50 (5k)	15 (1.5k)	52.9	53.3	N/A	N/A
Model Selection	1398	419	5k	1.5k	17.2	28.2	N/A	N/A
Structure Refinement	664	996	250	75	18.4	18.4	0.18	0.31
CDRH3 Design	7509	2275	250 (750)	75 (750)	2.0	3.8	12.0	12.9
Side Chain Packing	189	195	750	750	38.4	44.6	N/A	N/A
Structure Refinement	1446	1545	750	750	17.2	20.7	0.47	0.51
Best Mutants Selection	1083	1149	750	750	17.4	20.6	N/A	N/A
**Total per iteration**	**4.9h**	**2.2h**	**750**	**750**	**17-52**	**18-54**	**0.2-27**	**0.3-27**
Complete Pipeline (10 iter)	**24.7h**		**750**		**17-52**		**0.2-27**	

S4 Evaluation of nanobody *de novo* design and optimization

S4.1 Differential and relative binding free energy trends

NanoDesigner was applied in two distinct scenarios, using two different CDR generation methods (DiffAb and ADesigner), with analysis conducted on one fold. The plots in Figure S5, illustrate the comparison of average predicted ΔG (free energy change) and $\Delta\Delta G$ (change in binding energy) values over ten iterations across different NanoDesigner configurations. The left panels correspond to the optimization scenario, where the objective function of the algorithm focuses on improving binding affinity compared to a reference complex. The right panels represent the *de novo* scenario, where the algorithm optimizes the absolute binding energy without reference to a pre-existing complex.

In both scenarios, a general trend toward more negative values is observed over the ten iterations, indicating improved binding affinity and energy stability as the process progresses. The boxes show the distribution of the data, with whiskers highlighting the variability across iterations. ADesigner (purple) performs better in the optimization scenario, achieving more negative values consistently, while DiffAb (orange) shows stronger performance in the *de novo* scenario, suggesting it is more effective for generating novel designs without relying on a reference complex.

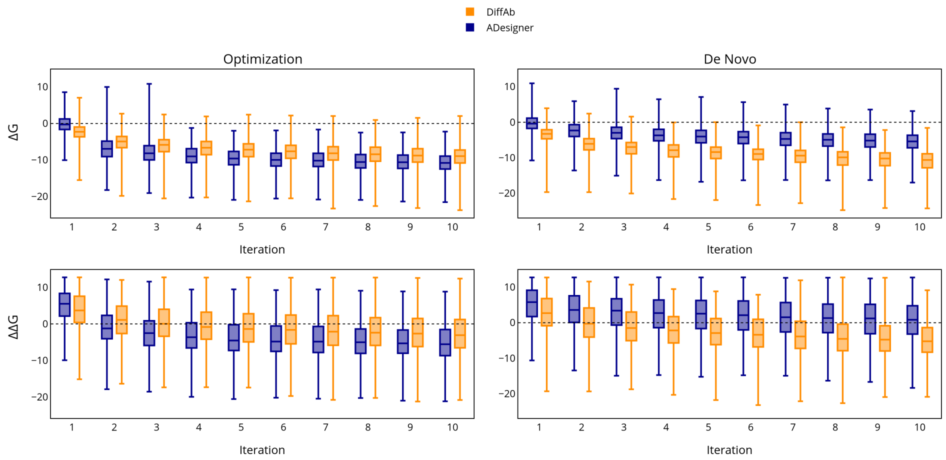

Fig. S5: Differential and relative binding free energy across iterations, results from NanoDesigner in the *de novo* and optimization scenarios using two CDR generation methods. Analysis was conducted on one fold from the optimal training configuration using the dataset combining antibodies and nanobodies, clustered by antigen sequence similarity 60%.

S4.2 Application of NanoDesigner on specific test cases

We applied NanoDesigner on three specific test cases; the test cases and the epitopes used are shown in Table S5. The epitopes together with the positional information of the three CDR loops were used as input.

Table S5: Inputs used for three NanoDesigner test cases. Epitope positional information for mNeonGreen (PDB:5LRT) and KRAS (PDB:4OBE) was determined using sequence data provided by domain experts. For HER2 (PDB:8PWH), epitope residue positions were obtained from the antibody-bound state using dSASA analysis.

Source PDB		User Epitope Sequence Input	Epitope Extracted Positional Information
**Nanobody**	**Antigen**		
6LR7	4OBE	NHFVDEYDPTIEDSYR	Chain A: (26, 'N'), (27, 'H'), (28, 'F'), (29, 'V'), (30, 'D'), (31, 'E'), (32, 'Y'), (33, 'D'), (34, 'P'), (35, 'T'), (36, 'I'), (37, 'E'), (38, 'D'), (39, 'S'), (40, 'Y'), (41, 'R')
		TAGQEEYSAMRDQYMRTGE	Chain A: (58, 'T'), (59, 'A'), (60, 'G'), (61, 'Q'), (62, 'E'), (63, 'E'),(64, 'Y'), (65, 'S'), (66, 'A'), (67, 'M'), (68, 'R'), (69, 'D'), (70, 'Q'), (71, 'Y'), (72, 'M'), (73, 'R'), (74, 'T'), (75, 'G'), (76, 'E')
6LR7	5LRT	NLKSTKGDLQF	Chain A: (38, 'N'), (39, 'L'), (40, 'K'), (41, 'S'), (42, 'T'), (43, 'K'), (44, 'G'), (45, 'D'), (46, 'L'), (47, 'Q'), (48, 'F')
		TTGNGKRYR	Chain A: (160, 'T'), (161, 'T'), (162, 'G'), (163, 'N'), (164, 'G'), (165, 'K'), (166, 'R'), (167, 'Y'), (168, 'R')
7EOW	8PWH	DQCVACAHYKDPPFCVARCP-SGVKPDLSYMPIWKFPDEEGACQP	Chain E: (583, 'K'), (555, 'F'), (603, 'P'), (579, 'P'), (592, 'W'), (569, 'K'), (572, 'P'), (582, 'V'), (570, 'D'), (560, 'D'), (557, 'P'), (571, 'P'), (561, 'Q'), (585, 'D'), (558, 'E'), (602, 'Q')
		LHCPALVTYNTDTFESMPNP-EGRYTFGASCVTACPYNYLSTDV	Chain E: (295, 'L'), (329, 'R'), (252, 'Y'), (255, 'D'), (257, 'F'), (284, 'T'), (311, 'K'), (285, 'D'), (235, 'H'), (128, 'K'), (248, 'A'), (294, 'P'), (297, 'N'), (268, 'T'), (296, 'H'), (290, 'T'), (286, 'V'), (236, 'F'), (245, 'H'), (254, 'T')

S4.2.1 Multi-CDR Design using DiffAb

Our dataset analysis revealed that all three CDRs are highly involved in binding, with consistent participation from CDRH1 and CDRH2 and more variable but frequently dominant contributions from CDRH3 (Figure S1, panel b; Table S1). While CDRH3 exhibits substantially greater structural diversity in both length and sequence composition compared to the more constrained CDRH1 and CDRH2 regions (Figure S2), the complementary nature of all three CDRs in antigen recognition necessitates their joint optimization during design. Therefore, we trained DiffAb, the best-performing method identified in our benchmarking analysis (Section S2.1), using the optimal

configuration with our combined nanobodies and antibodies dataset clustered at 60% antigen sequence similarity. This comprehensive training approach enables our NanoDesigner framework to also be further tested on a multi-cdr design setting.

Table S6: Performance of the DiffAB tool in the simultaneous design of nanobody CDRHs. The model was trained on both nanobodies and antibodies, with antigen sequence similarity set at 60%

Metric	DiffAb
AAR H1 ↑	0.397
AAR H2 ↑	0.426
AAR H3 ↑	0.240
RMSD ↓	4.633
RMSD CDRH1 ↓	2.546
RMSD CDRH2 ↓	1.729
RMSD CDRH3 ↓	3.855
TMscore ↑	0.815
LDDT ↑	0.677
ΔG ↓	13.058
$\Delta\Delta G$ ↓	19.341
Clashes ↓	17
DockQ ↑	0.180
Success Rate % ↑	14.5

S4.3 Sequence diversity of generated nanobodies

To assess the ability of each CDR design method — ADesigner, dyMEAN, and DiffAb — to generate diverse designs across different binding interfaces, we compared the sequence diversity of their CDRH3 nanobody designs. For consistency, all tools used the same set of docked models during inference, i.e., we did not use the NanoDesigner workflow but the methods directly. We generated sequence logos using the Logomaker library [9], where the x-axis represents the length of the CDRH3 sequences derived from PDB structures, and the y-axis shows the number of models included in the analysis. For illustrative purposes, we show a small subset of the generated CDR loops.

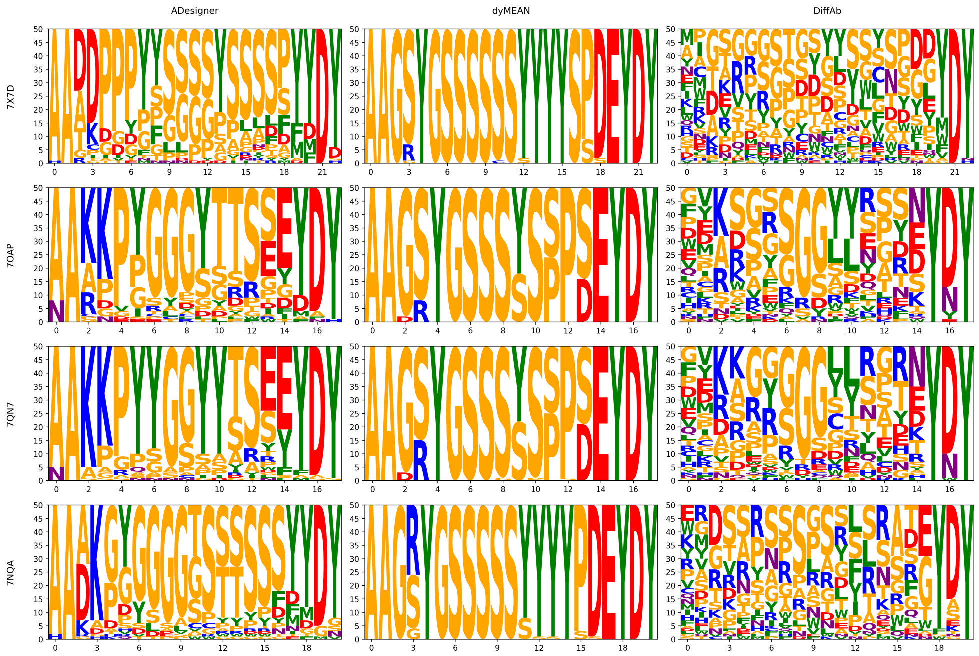

Fig. S6: CDRH3 sequence diversity of designed CDRH3 loops across methods for four cases. For entry `PDB:7OAP`, the nanobody referred to as "C1 nanobody" in the Protein Data Bank was used as input for the NanoDesigner process.

As shown in Figure S6, dyMEAN produced nearly identical sequences, indicating potential overfitting and limited generative capacity. ADesigner generated more diverse sequences but tended to include repetitive amino acids. DiffAb exhibited the highest sequence variability, indicating a broader exploration of the design space. Statistical analysis confirms these visual observations, revealing significant differences in sequence diversity across the three methods (ANOVA: $F = 157.04$, $p < 0.0001$ for position entropy; $F = 243.81$, $p < 0.0001$ for sequence uniqueness). DiffAb demonstrated significantly higher amino acid diversity at each position (mean entropy= 2.93) compared to both ADesigner (2.12, $p < 0.0001$) and dyMEAN (1.16, $p < 0.0001$). Most notably, DiffAb generated completely unique sequences (uniqueness ratio= 1.0), while ADesigner produced moderate redundancy (0.55) and dyMEAN showed high sequence repetition (0.13). These differences suggest distinct strategies for exploring the CDRH3 sequence space: DiffAb appears to maximize exploration, ADesigner balances exploration with exploitation of known motifs, and dyMEAN converges more strongly on a smaller set of sequence solutions. Such diversity patterns may impact each method's ability to discover novel, functional antibody designs, with DiffAb potentially offering greater coverage of the design space at the cost of including more non-functional sequences, while dyMEAN's more conservative approach might yield fewer but potentially more reliable designs.

References

[1] Dunbar, J., Krawczyk, K., Leem, J., Baker, T., Fuchs, A., Georges, G., Deane, C.M.: Sabdab: the structural antibody database. Nucleic Acids Research **42**(D1), 1140–1146 (2014). [Online]. Available: https://doi.org/10.1093/nar/gkt1043

[2] Berman, H.M., Westbrook, J., Feng, Z., Gilliland, G., Bhat, T.N., Weissig, H., Shindyalov, I.N., Bourne, P.E.: The protein data bank. Nucleic Acids Research **28**(1), 235–242 (2000) https://doi.org/10.1093/nar/28.1.235

[3] Ribeiro, J., Ríos-Vera, C., Melo, F., Schüller, A.: Calculation of accurate interatomic contact surface areas for the quantitative analysis of non-bonded molecular interactions. Bioinformatics **35**(18), 3499–3501 (2019)

[4] Al-Lazikani, B., Lesk, A.M., Chothia, C.: Standard conformations for the canonical structures of immunoglobulins. Journal of Molecular Biology **273**(4), 927–948 (1997) https://doi.org/10.1006/jmbi.1997.1354

[5] Steinegger, M., Söding, J.: Mmseqs2 enables sensitive protein sequence searching for the analysis of massive data sets. Nature Biotechnology **35**(11), 1026–1028 (2017). [Online]. Available: https://doi.org/10.1038/nbt.3988

[6] Jin, W., Wohlwend, J., Barzilay, R., Jaakkola, T.: Iterative refinement graph neural network for antibody sequence-structure co-design. arXiv preprint arXiv:2110.04624 (2021)

[7] Kong, X., Huang, W., Liu, Y.: Conditional antibody design as 3d equivariant graph translation. arXiv preprint arXiv:2208.06073 (2022). [Online]. Available: https://arxiv.org/abs/2208.06073

[8] Schymkowitz, J., Borg, J., Stricher, F., Nys, R., Rousseau, F., Serrano, L.: The foldx web server: an online force field. Nucleic Acids Research **33**(Web Server issue), 382–388 (2005)

[9] Tareen, A., Kinney, J.B.: Logomaker: beautiful sequence logos in python. Bioinformatics **36**(7), 2272–2274 (2020) https://doi.org/10.1093/bioinformatics/btz921
